# Aging and Curvature Discrimination from Static and Dynamic Touch

**DOI:** 10.1371/journal.pone.0068577

**Published:** 2013-07-02

**Authors:** J. Farley Norman, Astrid M. L. Kappers, Jacob R. Cheeseman, Cecilia Ronning, Kelsey E. Thomason, Michael W. Baxter, Autum B. Calloway, Davora N. Lamirande

**Affiliations:** 1 Department of Psychology, Western Kentucky University, Bowling Green, Kentucky, United States of America; 2 Center for the Study of Lifespan Development, Western Kentucky University, Bowling Green, Kentucky, United States of America; 3 Faculty of Human Movement Sciences, Vrije Universiteit, Amsterdam, The Netherlands; Emory University, United States of America

## Abstract

Two experiments evaluated the ability of 30 older and younger adults to discriminate the curvature of simple object surfaces from static and dynamic touch. The ages of the older adults ranged from 66 to 85 years, while those of the younger adults ranged from 20 to 29 years. For each participant in both experiments, the minimum curvature magnitude needed to reliably discriminate between convex and concave surfaces was determined. In Experiment 1, participants used static touch to make their judgments of curvature, while dynamic touch was used in Experiment 2. When static touch was used to discriminate curvature, a large effect of age occurred (the thresholds were 0.67 & 1.11/m for the younger and older participants, respectively). However, when participants used dynamic touch, there was no significant difference between the ability of younger and older participants to discriminate curvature (the thresholds were 0.58 & 0.59/m for the younger and older participants, respectively). The results of the current study demonstrate that while older adults can accurately discriminate surface curvature from dynamic touch, they possess significant impairments for static touch.

## Introduction

It has been known for more than 20 years that aging has detrimental effects on performance for simple tactile tasks. For example, tactile acuity deteriorates markedly with increases in age [1]–[5]; older adults, when performing a tactile grating orientation task, possess thresholds that are more than double (2.4 times higher than) those of younger adults in their twenties [2]. In addition, significant age-related differences in performance have been observed in a tactile letter identification task [6]. Given that these age-related differences in tactile acuity do exist, it is an interesting fact that older adults can perform as well as younger adults when they are asked to haptically discriminate [1] or estimate [2] solid object shape. In Experiment 1 of Norman et al. [1], younger and older adults haptically explored two solid objects on any given trial (bell peppers, Capsicum annuum) and were required to discriminate whether their shapes were the same or different. Their older participants performed as well as the younger participants despite the fact that they were more than 50 years older (on average). In Experiments 1 and 2 of Norman et al. [2], younger and older adults were asked to estimate the shape of quadric surfaces using either their entire hand or just the tip of their index finger. In both cases (hand and fingertip), the older participants’ judgments of 3-D surface shape were as accurate and reliable as those of the younger participants.

In a series of investigations in the late 1990’s [7], [8], Pont, Kappers, and Koenderink evaluated static tactile and active haptic curvature discrimination. They used a set of blocks whose top surfaces were curved in either a convex or concave manner – the curvature magnitudes of the convex and concave circular arcs varied from 0.2 to 1.8/m. Pont et al. found that their participants’ curvature discrimination thresholds depended upon which part of the hand or fingers was used to feel the curved stimuli. The thresholds also varied as a function of the spatial extent over which the stimuli were touched. Pont et al. concluded that their participants’ static and dynamic perceptions of curvature were derived from differences in surface attitude/orientation (i.e., surfaces with higher curvature have greater changes in surface attitude/orientation and surfaces with lower curvature have smaller changes in surface attitude/orientation).

In everyday interactions with objects, we often perceive important properties about them (e.g., their shape, curvature, size, etc.) from immediate contact, when our hands and fingers come into actual physical contact with object surfaces [9]–[11]. This physical contact stimulates slowly- and rapidly-adapting sensory mechanoreceptors within the skin [12]–[14]; the resulting patterns of cutaneous activity eventually produce activation in a variety of areas within the cerebral cortex [15], leading to conscious awareness and perception. It is very important to note, however, that cutaneous activity resulting from simple contact is not the only source of information available to support the tactile perception of object shape. When we actively manipulate objects using a variety of exploratory procedures [16], [17], this leads to activation of not only cutaneous receptors within the skin, but also results in the stimulation of muscle and joint receptors. The additional sensory and proprioceptive information made possible by active touch has been shown to enhance shape perception [18]–[22]. It has also been demonstrated that kinesthetic information alone [23], without any cutaneous activity at all, can be sufficient to permit the perception of shape [24]–[27]. For example, experimenters in the study by Magee and Kennedy [27] guided blindfolded participants’ fingers along the outlines of drawings of familiar objects; even though there was no cutaneous information at all (the participants’ fingers were not allowed to touch the drawings), the participants were able to successfully identify the depicted objects.

From the previous review of the literature, it is clear that shape perception can occur from static touch [7], [9]. It is also clear that active object manipulation [17]–[22] can facilitate shape perception. The kinesthetic information that accompanies active manipulation is an important source of information in its own right, apart from the shape-related information detected and transmitted by cutaneous receptors in the skin. Tactile acuity is conventionally measured in a static, passive manner (e.g., by the application of a tactile grating to an immobilized participant’s fingertip). Because aging has been shown to lead to significant deteriorations in tactile acuity, it is likely that older adults will also exhibit a reduced ability to discriminate curvature from static touch in the current experiments (because both static shape and tactile acuity tasks, such as tactile grating orientation discrimination, depend upon cutaneous input). If an age-related deficit in discriminating curvature from static touch is observed in the current experiments, it will not necessarily occur for dynamic touch (because older adults may be able to compensate by relying on the kinesthetic information that accompanies their own exploratory movements). The purpose of the current set of experiments was to explore this issue and compare younger and older adults’ abilities to discriminate surface curvature when they employ static and dynamic touch.

## Experiment 1

### Methods

#### Participants and ethics

Eight older adults (mean age was 75.6 years, SD = 4.5; their ages ranged from 71 to 85 years) and 8 younger adults (mean age was 22.1 years, SD = 3.0; their ages ranged from 20 to 29 years) participated in the experiment. The participants were either students at Western Kentucky University or were recruited from the local community (Warren County, Kentucky); three of the younger participants were coauthors (ABC, DNL, KET). All participants gave written consent prior to participation in the experiment. All of the participants (except the three coauthors) were naïve and unaware of the purposes of the experiment. The experiment was approved by the Western Kentucky University Human Subjects Review Board.

#### Apparatus

The order of presentation of the experimental stimuli was randomly determined for each participant by an Apple MacBook computer. The participants’ responses were entered into the computer for later analysis.

#### Experimental stimuli

**Figure 1:**
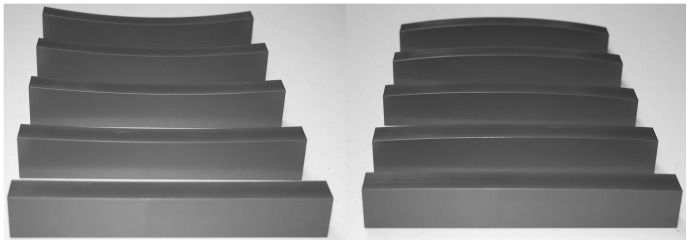
Photographs of ten of the stimulus objects. The objects with concave upper surfaces are shown on the left, while those with convex upper surfaces are presented on the right. The surface curvatures increase from 0.2 m^−1^ (located at the bottom) to 1.8 m^−1^ (located at the top).

The stimulus objects were the same as those used by Pont et al. [7], [8]; the curved blocks were 20 cm long, 2 cm wide, and approximately 5 cm tall. Their top surfaces were curved either in a convex or concave circular arc (see Figure 1); the curvature magnitudes ranged from 0.2 to 2.2/m. The curved blocks were made from PVC (polyvinyl chloride) using a computer-controlled milling machine. In addition to the surface curvature task, we evaluated the participants’ tactile acuity using tactile gratings (JVP Domes, Stoelting, Inc.; [28]). In particular, we used a set of tactile gratings where the groove widths ranged from 6 to 0.5 mm (6, 5, 4, 3, 2, 1.5, 1.2, 1.0, 0.75, & 0.5 mm).

#### Procedure

**Figure 2:**
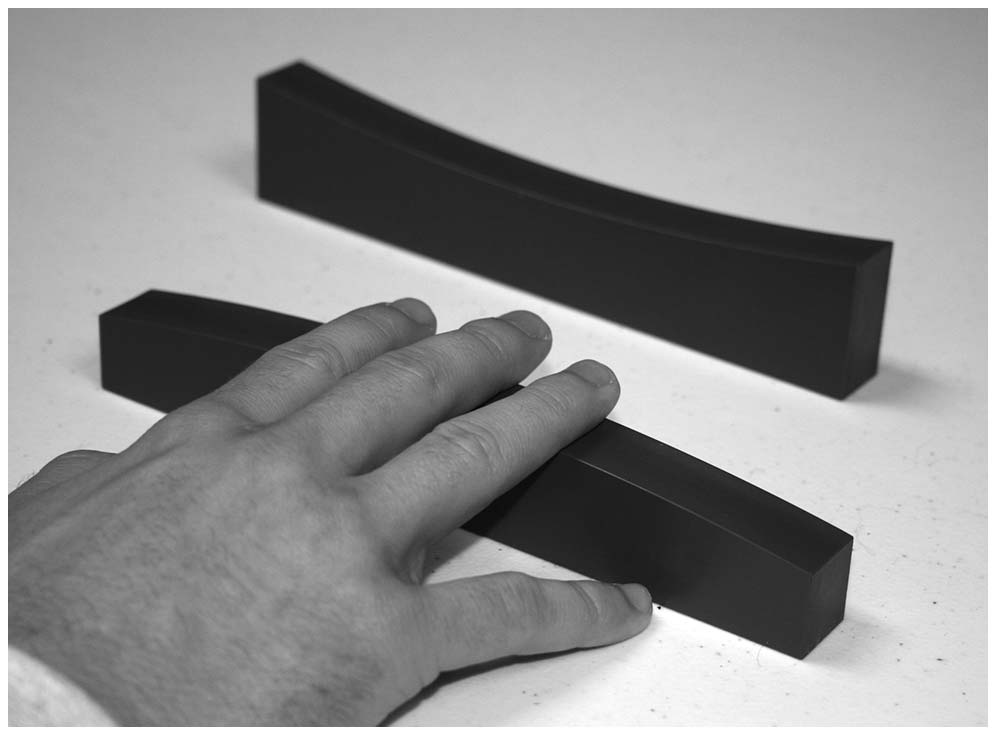
A photograph demonstrating how the participants used static touch when making a judgment in Experiment 1.

The procedures for the curvature discrimination task were similar to those of Pont et al. [7], [8]. The participants reached through an occluding curtain to feel the upper surface of the experimental stimuli. They used their three middle fingers to touch the upper surface of the curved blocks (see Figure 2); once contact was made with the block, the participants were not allowed to move their fingers (i.e., they used static touch). This was analogous to condition “6 normal” of Pont et al. [7], [8]. Goodwin et al. [9] found that the least amount of curvature that could be detected with a single stationary fingertip was 5.15/m (4.9/m for convex curvatures & 5.4/m for concave curvatures). In order to obtain the best performance (i.e., the lowest thresholds) for discriminations of static curvature, our participants therefore needed to use multiple fingertips simultaneously to sample the curvature of the blocks. The participants’ task on each trial was to judge whether any given stimulus block was convex or concave. The participants were given an unlimited amount of time to touch the experimental stimuli; most judgments, however, were made within two to three seconds (it is not surprising that the participants made their judgments rapidly; Srinivasan & LaMotte [12] demonstrated that there is considerable adaptation in the responses of cutaneous sensory receptors within one second of the application of a touch stimulus, see their Figure 3).

**Figure 3:**
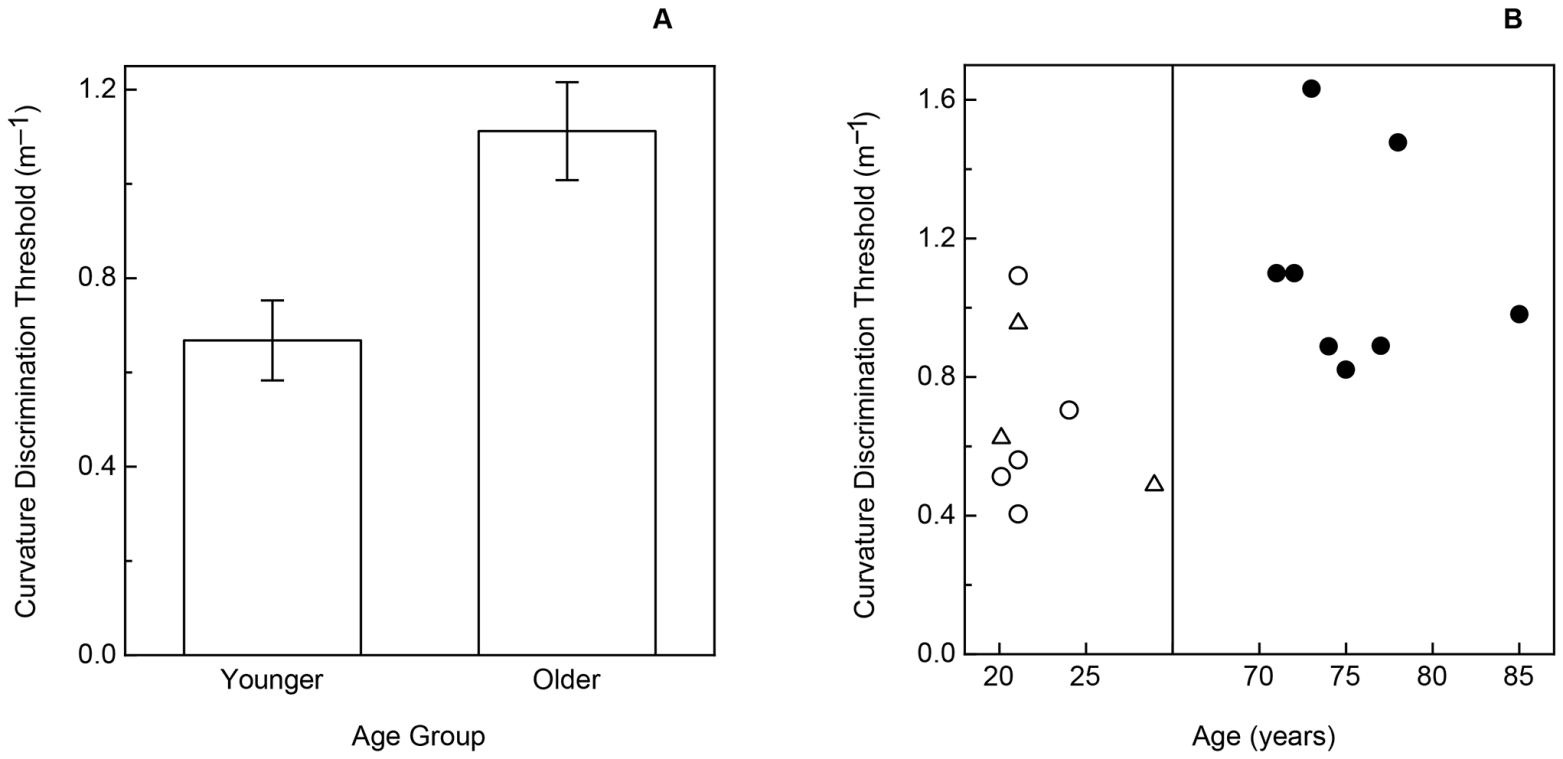
Experimental results for static curvature discrimination. A: The participants' overall curvature discrimination thresholds are plotted separately for each age group. The error bars indicate ± one SE. B: The individual younger and older participants' curvature discrimination thresholds are plotted as a function of age. The younger coauthors’ thresholds are depicted with open triangles, while thresholds for the younger non-authors are presented using open circles. The older participants’ thresholds are indicated by the filled circles.

An experimental session began with participants judging a block of 40 trials (20 trials with convex blocks & 20 trials with concave blocks, all presented in a random order) at the highest curvature (which was either 2.2 or 1.8/m). Pilot testing revealed that older adults were less sensitive to curvature; because of this, their testing began with blocks that possessed a curvature of 2.2/m. Succeeding blocks of 40 trials with lower curvatures (e.g., 1.4, 1.0, 0.6, & 0.2/m) were run, until the participants’ curvature discrimination performance dropped below a d’ value of 1.35. Once we had found curvature values that produced performance above and below each participant’s discrimination threshold (i.e., those curvatures that produced d’ values above and below 1.35), linear interpolation was used to calculate the final threshold estimate.

Similar procedures were used to measure the participants’ tactile acuity. Tactile gratings were briefly (approximately 1.0 seconds) applied manually [2], [28]–[30] to the distal fingerpad of the index finger; the grooves of the gratings were oriented either parallel or perpendicular to the long axis of the finger. The participants’ task was to judge (without vision) whether the grating’s orientation was parallel or perpendicular. Successive blocks of 40 trials (20 parallel trials & 20 perpendicular trials, all in a random order) with decreasing groove width were run, until each participant’s grating orientation discrimination performance dropped below a d’ value of 1.35. The threshold estimate was then calculated in an identical manner to that obtained for the surface curvature discrimination task. The initial groove width used for the younger participants was 3 mm. Larger initial groove widths (4 to 6 mm) were needed to determine thresholds for the older participants, because of the well known age-related deterioration in tactile acuity [1]–[5].

### Results and Discussion

**Figure 4:**
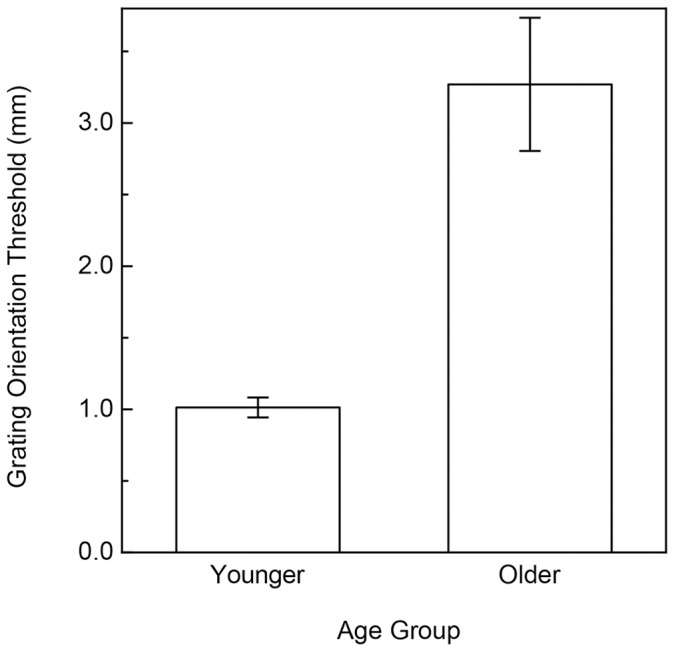
Experimental results (Tactile Acuity). The participants' grating orientation thresholds are plotted separately for each age group. The error bars indicate ± one SE.

The older and younger participants’ results are shown in Figures 3 and 4 for the curvature and grating orientation discrimination tasks, respectively. As is readily evident, there were significant effects of age for both tasks (curvature discrimination: t(14) = 3.3, p<.006, 2-tailed; grating orientation discrimination: t(14) = 4.8, p<.001, 2-tailed). On average, the older participants’ thresholds were 66.4 percent higher than those of the younger participants for the surface curvature discrimination task, and were more than three times (3.23 times) higher than those of the younger participants for the grating orientation task. The thresholds for the younger participants who were coauthors (ABC, DNL, KET) were not significantly different from the non-author younger participants for both tasks (curvature discrimination: t(6) = 0.2, p = .87; grating orientation discrimination: t(6) = 1.9, p>.1). As can be seen in Figures 3 and 4, the effect sizes were large (Cohen’s d was 1.66 & 2.98 for the curvature and grating orientation discrimination tasks, respectively). Given the effect sizes and the number of participants in each age group, the resulting power (for a 2-tailed test, α = .05) was 0.91 and 0.99 for the curvature and grating orientation discrimination tasks, respectively. This means, for example, that given the size of the obtained effects and the magnitude of the inter-participant variability, that we had a 91 to 99 percent chance of detecting the effects with our chosen sample size.

Given that all of the participants performed both tactile judgments (curvature discrimination & grating orientation discrimination), we decided to evaluate whether there is any relationship between the performances obtained for these two tasks. We found that there was no significant correlation (Older participants: Pearson r = −0.2, r^2^ = 0.04, p = .62; Younger participants: Pearson r = 0.004, r^2^<.001, p = .99). Thus, if any given participant performs well for curvature discrimination, it tells us nothing about their tactile acuity; conversely, high tactile acuity does not reveal anything about a participant’s ability to discriminate surface curvature.

## Experiment 2

The results of Experiment 1 demonstrate that older adults can reliably discriminate surface curvature. However, their ability to detect differences in static curvature is impaired relative to the abilities of younger adults (see Figure 3). It is important to keep in mind that this age-related impairment for static touch might not necessarily occur for dynamic touch. This is because during active touch kinesthetic and proprioceptive information is available in addition to cutaneous information from the hand and fingers [18], [24]–[26]. It is conceivable that older adults could compensate for the reduction in cutaneous information about static curvature by taking best advantage of the proprioceptive information that occurs during active haptic manipulation. The purpose of Experiment 2 was to examine this possibility by allowing our participants to actively feel the experimental stimuli while discriminating surface curvature.

## 

### Methods

#### Participants and ethics

The participants were 14 naïve adults, none of whom had participated in Experiment 1. Seven of the participants were 66 years of age or older (mean age was 71.9 years, SD = 3.4; ages ranged from 66 to 77 years), while the remaining seven participants were 25 years of age or younger (mean age was 22.0 years, SD = 1.4; ages ranged from 21 to 25 years). The participants were either students at Western Kentucky University or were recruited from the local community (Warren County, Kentucky). All participants gave written consent prior to participation in the experiment. The experiment was approved by the Western Kentucky University Human Subjects Review Board.

#### Apparatus

The apparatus was the same as that used in Experiment 1.

#### Experimental stimuli

The stimulus objects were the same as those used in Experiment 1.

#### Procedure

The basic procedure and task was identical to that used in Experiment 1. The participants would reach through an occluding curtain and feel a single stimulus block on any given trial. In this experiment, however, the participants would actively feel the middle 10-cm section of each curved block’s upper surface with a single index finger. As in Experiment 3 of Pont et al. [8], the participants’ finger movements were limited (i.e., restricted to 10 cm) by an aperture. Once again, the participants had an unlimited amount of time to feel each curved surface and determine whether it was convex or concave; most of the judgments were made within two to three seconds.

The remainder of the procedures for the curvature discrimination and tactile acuity tasks were identical to those used in Experiment 1. Successive blocks of 40 trials were once again run for both tasks, with decreasing curvature and groove width, until performance dropped below a d’ value of 1.35. The final threshold estimates were calculated in the same manner as was done in Experiment 1.

### Results and discussion

**Figure 5:**
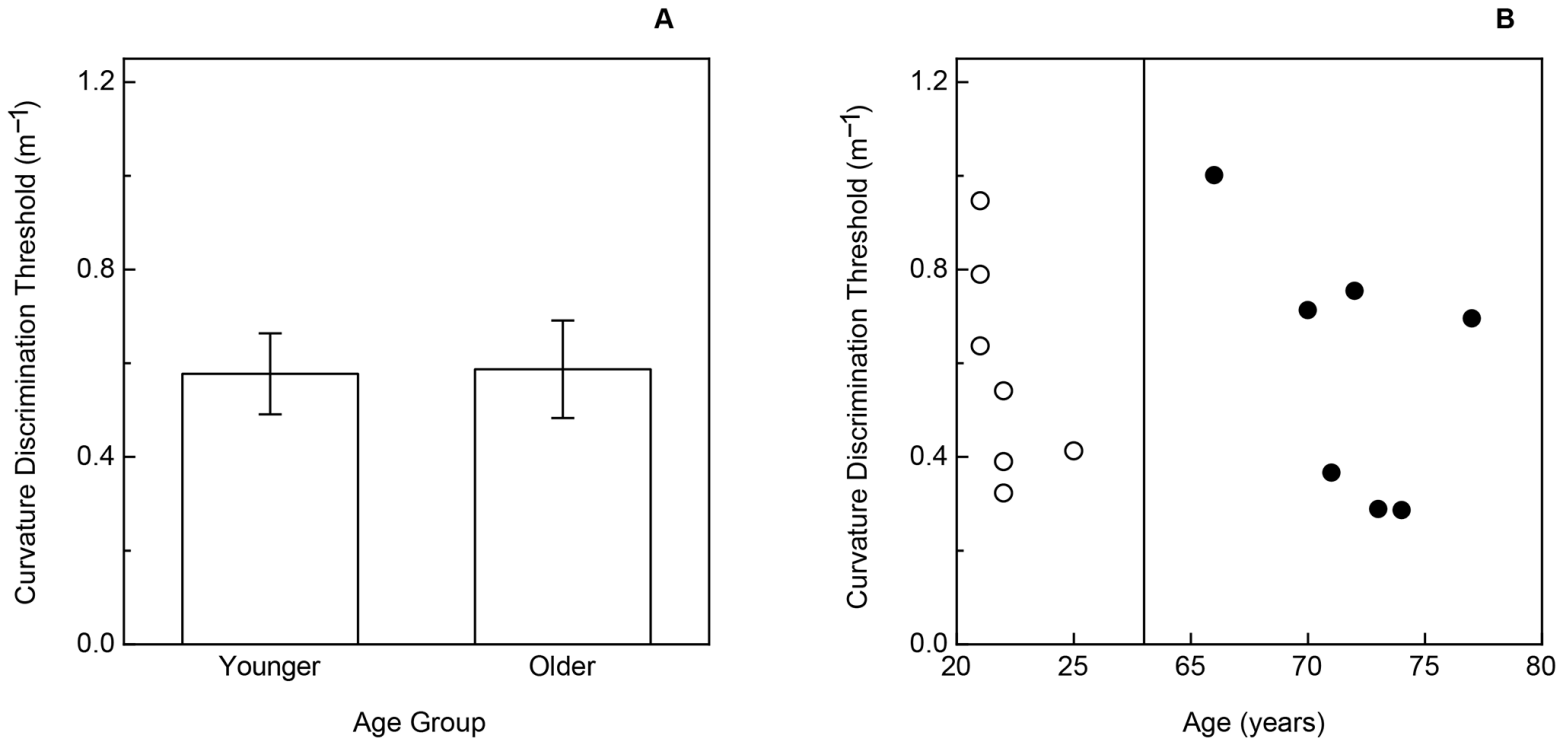
Experimental results for dynamic curvature discrimination. A: The participants' overall curvature discrimination thresholds are plotted separately for each age group. The error bars indicate ± one SE. B: The individual younger (open circles) and older participants' (filled circles) curvature discrimination thresholds are plotted as a function of age.

**Figure 6:**
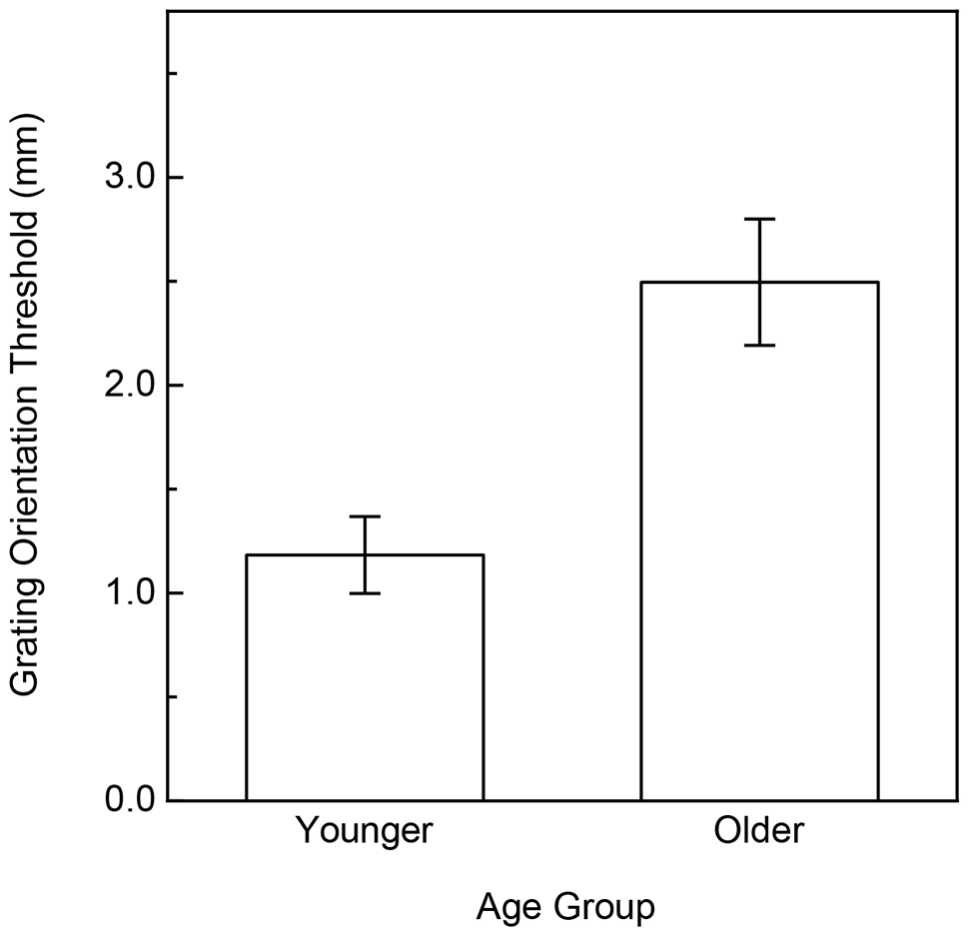
Experimental results (Tactile Acuity). The participants' grating orientation thresholds are plotted separately for each age group. The error bars indicate ± one SE.

The results of the dynamic curvature discrimination task are shown in Figure 5, while the results for the tactile grating orientation task are shown in Figure 6. Once again, there was a significant effect of age upon tactile acuity (t(12) = 3.7, p = .003, 2-tailed): the older participants’ grating orientation thresholds were more than double those of the younger participants (see Figure 6). However, the pattern of results obtained for the curvature discrimination task was unlike that observed in Experiment 1. In this experiment, there was no effect of age (see Figure 5) upon the participants’ curvature discrimination thresholds (t(12) = 0.07, p = .94, 2-tailed). The individual participants’ curvature discrimination thresholds are shown in the right panel of Figure 5. One can see from this figure that the distributions for the younger and older participants overlap completely, and that within the group of older participants themselves, increases in age did not make any difference – i.e., performance for the 77 year-old participant was just as good as (actually better than) that exhibited by the 66 year-old participant. The difference between the average curvature discrimination thresholds of the younger and older participants (left panel of Figure 5) was 0.01/m; a power analysis revealed that a total of 25,002 participants (12,501 older participants and 12,501 younger participants) would be needed to have a 90 percent chance of detecting a difference this small. It is readily apparent that even if this difference (curvature discrimination thresholds of 0.587 vs. 0.577/m) did exist in the general population, it is of no practical or meaningful importance.

A 2-way between-subjects analysis of variance (ANOVA) was conducted to compare the results obtained for static (Experiment 1) and dynamic (Experiment 2) touch. The results of the ANOVA revealed that there were main effects of both touch type (static vs. dynamic, F(1, 26) = 10.3, p = .003, partial 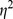 = .29) and age (F(1, 26) = 5.6, p<.03, partial 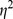 = .18). However, it is readily evident from a comparison of Figures 3 and 5 that the large effect of age obtained for static touch disappeared when the participants dynamically explored the curvature of the experimental stimuli (i.e., there was a significant interaction: F(1, 26) = 5.1, p = .03, partial 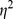 = .17).

## General Discussion

The results of Experiment 1 clearly indicate that aging has significant and large effects upon the ability to discriminate surface curvature by static touch (Figure 3); this large effect of age resembles the well-known deterioration that occurs in tactile acuity [1]–[6](also see Figure 4). Apparently, aging negatively affects performance for multiple static tactile tasks. However, it is interesting in this context to note that performance for the current two static tasks (the grating orientation task used to evaluate tactile acuity and static surface curvature discrimination) do not correlate to any appreciable degree, either for older or younger adults.

In comparison to Experiment 1, the participants’ performance improved (thresholds were lower, compare Figures 3 and 5) when information obtained by dynamic touch was available – this was especially true for the older participants. Many studies have similarly shown that active touch leads to better shape perception than passive touch [17]–[22]. It is also clear from the results of Experiment 2 that there was no significant effect of age upon the ability to discriminate surface curvature from dynamic touch (Figure 5). One possibility for the equal ability of older adults to dynamically perceive and discriminate surface curvature (despite the fact that older adults’ static curvature discrimination is impaired) is the availability of kinesthetic information during active/dynamic exploration [23]. It is well known that the kinesthetic and proprioceptive information that accompanies hand and arm movements permits the perception of object shape all on its own, apart from the information about shape provided by cutaneous receptors in the skin [18], [24]–[27]. While previous studies have found that kinesthetic and cutaneous inputs provide similar information enabling the recognition of raised-line drawings [31], [32], other studies have found kinesthetic information to predominate [25], [27]. For example, in one condition of a study by Magee and Kennedy [27], the experimenters guided participants’ hands along the outer contours of drawings of familiar objects (no cutaneous input). In another condition, the experimenters moved the contours of raised-line drawings underneath participants’ stationary fingertips (no kinesthetic input). The participants in this study who had access to kinesthetic information (but not cutaneous information) recognized many more depicted objects than those participants who only had access to cutaneous (but not kinesthetic) information. In the current study, the curvature discrimination performance of both older and younger participants improved in Experiment 2 when kinesthetic information (resulting from dynamic touch) was available. The magnitude of the improvement was larger, however, for the older participants; this larger improvement for the older participants allowed them to perform just as well as the younger participants (see Figure 5). If it was the kinesthetic information available in Experiment 2 that permitted the older participants to perform as well as the younger participants, it seems likely that we would probably also have obtained similar results (i.e., no effect of age for dynamic curvature discrimination) if we had removed cutaneous information altogether and required our participants to judge curvatures solely from kinesthetic input.

Given our current findings that the negative effects of increased age only exist for static touch (Figure 3) and do not occur for situations involving active touch (Figure 5), it is unlikely that the deficits found in the current study significantly impair many of the everyday activities of older adults.

## Conclusions

Aging is accompanied by substantive deficits in static, but not dynamic, surface curvature discrimination.
